# Rheology Impact of Various Hydrophilic-Hydrophobic Balance (HLB) Index Non-Ionic Surfactants on Cyclopentane Hydrates

**DOI:** 10.3390/molecules25163725

**Published:** 2020-08-15

**Authors:** Khor Siak Foo, Cornelius Borecho Bavoh, Bhajan Lal, Azmi Mohd Shariff

**Affiliations:** 1 Chemical Engineering Department, Universiti Teknologi Petronas, Seri Iskandar, Teronoh, Perak 32610, Malaysia; khorsiakfoo88@gmail.com (K.S.F.); bavohcornelius@gmail.com (C.B.B.); azmish@utp.edu.my (A.M.S.); 2 CO_2_ Research Centre (CO2RES), Universiti Teknologi Petronas, Perak 32610, Malaysia

**Keywords:** clathrate hydrates, surfactants, span, tween, cyclopentane, rheology

## Abstract

In this study, series of non-ionic surfactants from Span and Tween are evaluated for their ability to affect the viscosity profile of cyclopentane hydrate slurry. The surfactants; Span 20, Span 40, Span 80, Tween 20, Tween 40 and Tween 80 were selected and tested to provide different hydrophilic–hydrophobic balance values and allow evaluation their solubility impact on hydrate formation and growth time. The study was performed by using a HAAKE ViscotesterTM 500 at 2 °C and a surfactant concentration ranging from 0.1 wt%–1 wt%. The solubility characteristic of the non-ionic surfactants changed the hydrate slurry in different ways with surfactants type and varying concentration. The rheological measurement suggested that oil-soluble Span surfactants was generally inhibitive to hydrate formation by extending the hydrate induction time. However, an opposite effect was observed for the Tween surfactants. On the other hand, both Span and Tween demonstrated promoting effect to accelerate hydrate growth time of cyclopentane hydrate formation. The average hydrate crystallization growth time of the blank sample was reduced by 86% and 68% by Tween and Span surfactants at 1 wt%, respectively. The findings in this study are useful to understand the rheological behavior of surfactants in hydrate slurry.

## 1. Introduction

**Figure 1:**
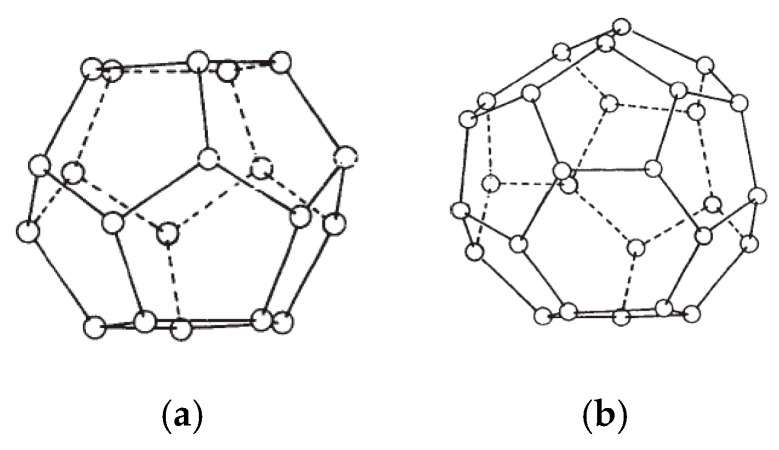
Type II hydrate structures of small cage and large cage. (**a**) Dodecahedron 12-sided polyhedron (small cage); (**b**) hexakaidecahedron 16-sided polyhedron (large cage).

Clathrate hydrates—also known as gas hydrates—are solid compounds that form when a suitable size substance (gas/liquid) is encapsulated in a 3-dimensional network water cage held together through hydrogen bonding [1,2,3]. The encapsulating substances are typically light hydrocarbons or small gaseous molecules such as methane, ethane, propane, nitrogen and carbon dioxide. The presence of these small substances known as hydrate formers stabilizes the gas hydrate structure by a weak van der Waals force. High pressure and low ambient temperature favor and enhance gas hydrate formation and stability [1,4]. Depending on the arrangement of the water molecules constructing the hydrate crystals network, hydrates structures can be classified as Type I and Type II—sometimes referred to as Structure I and Structure II. A third type of hydrate that also may be encountered is Type H (or Structure H), but its occurrence is much less common in natural environment. The structure of the Type II hydrates is significantly more complicated than that of the Type I hydrate. The Type II hydrates can be formed from two different types of cage. The small cage of Type II structure is a dodecahedron, a twelve-sided polyhedron where each face is a regular pentagon, whereas the structure of big cage has a hexakaidecahedron, a sixteen-sided polyhedron with twelve pentagonal faces and four hexagonal faces as shown in Figure 1.

Clathrate hydrates pose major operational threats to oil and gas flow assurance [5]. The increase of deep-water oil and gas production poses severe hydrate threats due to high pressure and low temperature conditions of multiphase fluid flow in subsea production wells and pipelines [6]. Hydrate formation in pipelines may cause economic and safety risks due to unplanned shutdown and high cost of removal [1]. The current conventional approaches to hydrate plug prevention is the use of chemical inhibitors [7,8,9,10,11,12,13,14,15,16]. However, thermodynamic inhibitors such as methanol and glycols have limitations of effectiveness and environmental concerns [17]. Thus, efforts have been shifted to manage hydrate formation via the use of low dosage hydrate inhibitors (LDHI), which include the use of anti-agglomerate low dosage hydrate inhibitor (AA-LDHI) [18,19,20,21,22]. This class of inhibitors generally prevent hydrate agglomeration and aid the transportation of hydrate as slurry [23]. Therefore, a critical understanding of the rheological properties of hydrate slurry in the presence of AA-LDHI is needed to efficiently manage hydrate formation in subsea facility design and operations.

It has been reported that cationic surfactants are effective anti-agglomerant low dosage hydrate inhibitor (AA-LDHI) [24,25]. These surfactants control hydrate growth by dispersing the hydrate crystals that form at water-hydrocarbon interface into discrete suspended particles without allowing them to adhere and agglomerate into big particles, so to remain in a flowable and pumpable multiphase stream. The adsorption of the cationic surfactant molecules onto the hydrate particles surface enhances the oil wetting characteristic of the hydrate particles so they become part of the hydrocarbon phase and agglomeration of the discrete hydrate particles is inhibited [25].

On the other hand, anionic surfactants promote hydrate formation by participating partly in the gas hydrate clathrate formation causing reduction in adhesion forces among hydrate molecules allowing for a larger particle surface area for hydrate growth [26]. It has also been proposed that some anionic surfactants such as sodium dodecyl sulfate (SDS) and linear alkyl benzene sulfonic acid (LABSA) accelerate hydrate growth through the formation of hydrophobic micro-domains within vicinity of the hydrate surface that increases gas concentration and intake rate for hydrate formation. There are substantial studies on the morphology of surfactants on hydrate inhibition, the rheological behavior of surfactants in hydrate inhibition is not well understood.

Recent morphology study has reported some non-ionic surfactants such as Span 80 and Tween 65 as potential additives to prevent hydrate formation. However, limited work has been done to understand the effects of non-ionic surfactant concentrations on hydrate formation and growth. In addition, their rheological behaviors have not been well studied in literature [27]. Depending on the molecular structure and characteristics, adsorption of non-ionic surfactants would alter the surface energy and wetting behavior of gas hydrate particles. This interaction could change the hydrate slurry rheology and potentially delay or promote the rate of gas hydrate formation. In addition, the effect of their concentration and HLB values on hydrate slurry is not fully known. Hence it is encouraging to test the rheological behavior of hydrates in the presences of non-ionic surfactants. In this study, the effect of Span and Tween surfactant on cyclopentane hydrate slurry is evaluated in detail at varying surfactant concentrations.

### 1.1. Cyclopentane as Hydrate Former

Many gas hydrate studies involve using liquid cyclopentane as hydrate former to test the performance of new compounds [27,28,29,30,31,32,33]. This compound could form hydrate crystals at atmospheric pressure mode with sufficient subcooling to overcome the difficulties in testing the rheological behavior of hydrate slurry at high pressure conditions [27]. Therefore, adopting this hydrate model system to represent real gas hydrate is considered practical and valid due to the low water solubility of the cyclopentane mimicking interaction of hydrocarbon gas presence in water. More important, cyclopentane hydrate is known to form a structure II (sII) hydrate which is one of the most common hydrate crystal structure found naturally [27].

**Figure 2:**
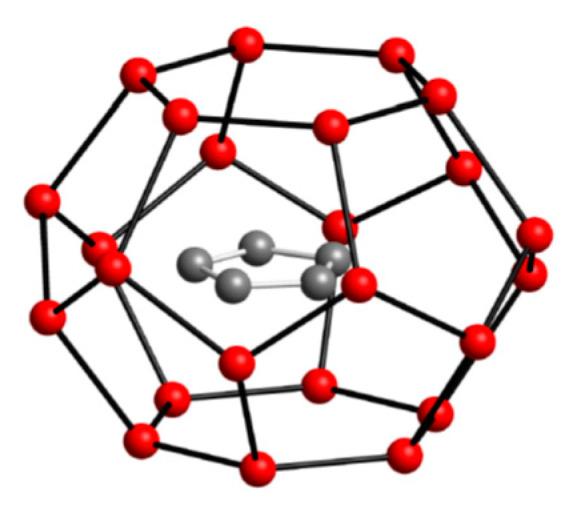
Cyclopentane hydrate. H_2_O molecules in red cage the guest hydrocarbon molecule inside.

The size and structure of cyclopentane lends itself to efficient space filling of structure II (sII) cavities, as shown by Figure 2. Cyclopentane is at the upper size limit for molecules that promote the formation of sII hydrates, but it can also form sH with helper molecules such as methane to stabilize the structure by filling in small cavities [1,4]. It was not until 2001 that Fan et al [30]. first confirmed cyclopentane can form gas hydrates in the absence of any helping-gas.

### 1.2. SPANS and TWEENS Surfactants

Spans and Tweens are non-ionic surfactants that are used as emulsifying agents in the preparation of stable emulsion systems for many applications [34]. They are also frequently used in varying combination proportions to produce desired emulsification stability to target different types of oil–water systems. Being non-ionic in nature, these surfactants have no inherit ionic interaction affinity and are widely compatible and stable in many fluid systems including fresh water, saline water, mild acids, alkaline and do not react with ionic ingredients and charged substances. Which makes them good candidates for hydrate-based applications. Spans are sorbitan esters that are manufactured from the dehydration of sorbitol through esterification reaction with fatty acids. The size and length of the fatty acid molecules used would decide the hydrophilic–hydrophobic balance (HLB) and the solubility of the surfactant in polar and nonpolar solvent. Further esterification with fatty acids results in polyesters [35]. The HLB value decreases with increasing degrees of esterification and size of the fatty acid molecules. Low HLB Spans favor solubility in lipophilic solvent to produce stable water-in-oil emulsion system [35].

On the other hand, Tweens are produced from ethoxylation of Spans to increase HLB range and enhance hydrophilic solubility to yield stable oil-in-water emulsion system [36]. Tweens are soluble or dispersible in water and dilute solutions of electrolytes. The solubility of Tweens in aqueous solution increases with the degree of ethoxylation. However, for a fixed degree of ethoxylation and esterification, aqueous solubility decreases as the size and molecular weight of the fatty acid increases. Spans derived from unsaturated and branded chain fatty acids act as effective water-in-oil emulsifiers. It is believed that the above stated properties of Span and Tween presents them as suitable inhibitors for gas hydrate management [28,29]. Therefore, to efficiently evaluation the effect of Span and Tween on hydrate formation, Span and Tween with different HLB values are selected and tested in this work. Furthermore, all the selected Span and Tween surfactants are tested at different concentrations.

### 1.3. Sorbitan Ester and Derivatives for Cyclopentane Hydrate Control

Delgodo-Linares et al. [37] evaluated the stability of water-in-oil emulsion from mineral oil 70 T and deionized water in the presence of surfactants of non-ionic Span 80 and anionic AOT (di-2-ethylhexylsulfosuccinate). Bottle testing in laboratory at ambient pressure showed that all the emulsion samples prepared in 1% and 5 wt% surfactants were stable against coalescence for at least one week. From the microscopy images of the emulsions, the average water droplet size ranges from 2 to 3 µm. Rheological measurements showed that the viscosity of the emulsion increased with increasing water cut. It was also observed that these emulsions showed shear thinning behavior above certain water cuts (30 vol% at 25 °C and 20 vol% at 1 °C). Differential scanning calorimetry (DSC) measurements showed that the emulsions prepared with 5 wt% surfactant with less than 50 vol% water were the most stable upon hydrate formation/dissociation. A further micromechanical force (MMF) measurements revealed that the presence of the surfactant mixture of Span 80 and AOT has little impact in the cohesion force of hydrate particles, although a change in hydrate surface morphology was observed. Autoclave cell experiments showed two different types of hydrate formation morphology; large hydrate chunks for the system without surfactant and loose hydrate slurry of the system with surfactant.

In addition, Liua et al. [38] studied the interaction between cyclopentane hydrate particles and water droplets using a micromechanical force apparatus [6]. This unique apparatus setup could provide direct measurements of the interaction force between two particles surface in contact while any morphology change in hydrate particles is observed and recorded using an inverted light microscope. The effects of contact time, sub-cooling temperature, contact area and the addition of Span 80 on the water droplet–hydrate particle interaction force were reported. The results indicated that longer contact time and higher sub-cooling lead to more water converting to hydrate, which increased the hydrate particle–water droplets interaction force significantly. The interaction force also increased with hydrate–water droplet contact area. The adsorption of Span 80 induced a morphologic change on the hydrate surface, where hair-like extrusions extended from the surface into the cyclopentane bulk phase. Addition of Span 80 after formation of a hydrate shell appeared to weaken interfacial connection/junction between surface crystals. Internal water then flowed out to contact the external cyclopentane, leading to rapid hydrate formation and consequently an irregular surface growth. Meanwhile, the presence of Span 80 on both the hydrate and water interfaces can form adsorption layers with stronger mechanical strength. This provides more stable interface again particle coalescence, thereby hindering the formation of water bridges. This postulated that Span 80 can prevent hydrate agglomeration.

The effects of hydrophilic–lipophilic balance (HLB) number for series of non-ionic sorbitan monoester on equilibrium condition of cyclopentane hydrate in water-in-oil emulsion was reported by Baek et al [31]. The performance of Span 20, Span 40, Span 60 and Span 80 to control formation of cyclopentane hydrate was compared for their effects on droplet size distribution and hydrate dissociation temperature using optical microscope and micro differential scanning calorimetry method. It was found that the mean size of emulsion droplets and the equilibrium temperature of cyclopentane hydrates was proportion to their HLB index. However, the variations of hydrate equilibrium temperature and emulsion size with the HLB index was practically eliminated with the addition of NaCl except for the case of Span 60. The equilibrium temperature of cyclopentane with sorbitan Span 80 and 3.5% NaCl was reduced from 4.75 °C to 5.5 °C for cyclopentane hydrate with water only. It was believed that the hydrophobic tails of the sorbitan monoester act as a physical barrier to interrupt the coalescence of emulsion droplets and reduced the hydrate dissociate temperature. In case of Span 60, unbalanced activity of the surfactant can be induced by a strong interaction between water and salt ions. However, the rheology of cyclopentane hydrates in the presence of the spans were not evaluated.

Karanjkar et al. [27] revealed the rheological properties of cyclopentane hydrate in a water-in-oil emulsion system. Span 80 was studied for its effect to render the effect of viscosity increased during cyclopentane hydrate formation [27]. Liquid cyclopentane hydrate slurry was prepared at atmospheric pressure from a water-in-oil emulsion by quenching it to a lower temperature at a fixed shear rate. It was observed that viscosity increased by several orders of magnitude indicating formation of hydrate on the dispersed water droplets and subsequent agglomeration. They attributed the observation to formation of a hairy and porous hydrate growth combined with enhanced agglomeration due to liquid bridges formed by wetted water films. The development of void space as water converted to hydrate within the hydrate structure network contributed to greater effective dispersed phase fraction that increased the viscosity dramatically. An observed dependence of the effective viscosity of the hydrate slurry on the degree of sub-cooling, with lower slurry viscosity obtained at higher sub-cooling. The possible anti-agglomerate like effect of high Span 80 concentrations, which led to a lower viscosity, was due to the availability of excess amount of surfactant in the oil phase which readily and efficiently adsorbed on the hydrate–oil interface and thus preventing a strong interaction between hydrate particles.

Recently, Dann and Rosenfeld [26] studied the hydrate inhibitive performance of Span 20, Span 80, Pluronic L31 and Tween 65 at 2 °C, using cyclopentane hydrate and deionized water. He observed morphology shift in crystallization from planar shell growth to conical growth in surfactants. Monitoring the internal pressure of a droplet undergoing planar hydrate crystallization in a hydrate-visualization cell provided a strong correlation of decreasing interfacial tension to the shrinking area of the water–cyclopentane interface. Hydrate growth rate reduced most by Tween 65 (at 0.15 g/100 mL) from 0.59 mm^2^/min to 0.068 mm^2^/min (of pure water), nearly an order of magnitude slower than that found for pure water at 0.590 mm^2^/min. High molecular weight (1845 g/mol) and HLB (10.5) contributed to a large energy of desorption at an interface and are believed to be the sources of Tween 65 hydrate inhibiting properties. They further suggested that hydrate morphology is affected by the type and concentration of surfactants. However, the rheological analysis of surfactants type and concentrations are not well understood and studied in literature. Most hydrate slurry rheology studies are limited to Span 80. There are few studies in open literature that employ rheological approach to study hydrate slurry behavior in the presence of surfactants. In particular, tween-based surfactants. In addition, understanding the effect of surfactants concentration on hydrate slurry is necessary to provided effect guidelines in their practical applications. Therefore, in this article, the rheological slurry of cyclopentane hydrate is evaluation in the presence of three series of Span and Tween surfactants at different concentrations.

## 2. Materials and Methods

### 2.1. Materials and Sample Preparation

The list of chemicals used in this work is tabulated in Table 1. They were used as supplied, without any further purification. The selection of Span and Tween surfactants was based on their HLB values. Allowing an effective evaluation on the impact of the surfactants to enhance the interfacial interaction between gas phase (hydrate former), water phase and solid hydrate particles. The effect of Span and Tween on cyclopentane hydrates were tested at a concentration range of 0.1 wt% to 1.0 wt%.

The tested sample mixture comprised of 50% volume of cyclopentane and 50% water at 25 °C. Representing a mixture of 9.0 mL cyclopentane mixed with 9.0 mL of deionized water in a 50-mL glass sample bottle and gently tilted upside-down 50 times. Slow-and-gentle mixing was applied only to well the mixture well but was not intended to produce a tight emulsion with the surfactant present. The sample with Span and Tween were prepared by first prepared the desired Span–Tween concentration (0.1 wt%–1.0 wt%). After which the cyclopentane and deionized water were used to dilute the Span and Tween surfactants, respectively before the mixtures were put together in the glass sample bottle.

### 2.2. Experimental Apparatus and Rheology Measurement Procedure

The rheological properties of semi-solid hydrate slurry were studied using HAAKE ViscotesterTM 550. The viscometer is designed with a rotational speed preset and measure the flow resistance of a slurry fluid. The torque maintaining the set speed is proportional to the fluid viscosity, so all final information on the viscosity, shear stress and the shear rate were obtained from the torque required, the set speed and the geometry factors for the applied sensor. The study was performed with a cylindrical concentric geometry. The temperature of the viscometer is controlled by an attached external water bath system. In each experimental run, the desired prepared sample mixture of water and cyclopentane (with or with Span/Tween) is loaded into the viscometer sample holder at a preset temperature of 15 °C. The temperature of the water bath is then lowered to the desired experimental temperature 2 °C at a rate of 0.21 °C/min, with the controlled shear rate of 50 s^−1^. This was done to allow cyclopentane hydrate to form. Due to the long probabilistic nature of cyclopentane hydrate formation, the sample mixtures were seeded at the interface at the experimental temperature about 60 min at the start of the experiment [27]. The time of seeding was taken as the time zero to hydrate formation. A sharp increase in the system viscosity indicated the onset of hydrate crystallization or formation as shown in Figure 2 [39]. Further developing and agglomeration of more hydrate particles continue to increase the slurry viscosity until a point that the amount of hydrate particle was substantial to completely jam and cease and rotational motion of the rotating spindle.

**Figure 3:**
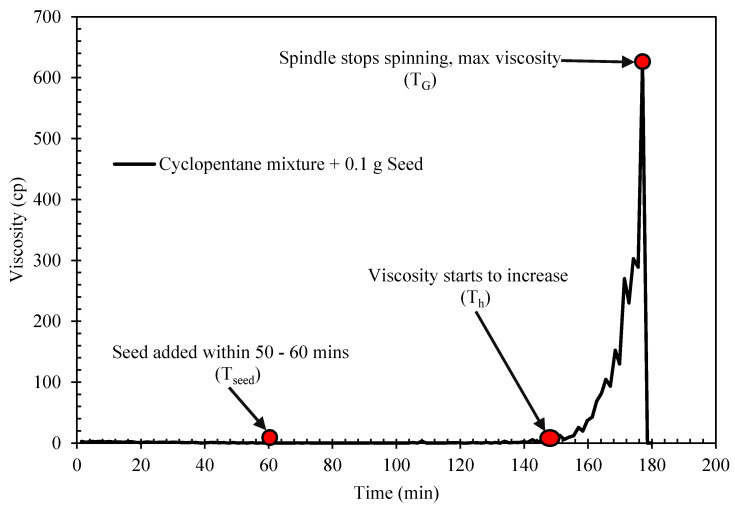
Typical hydrate rheology profile of cyclopentane mixture (Blank) with 0.1 g seed at 2 °C.

The hydrate induction time and crystallization/growth time were estimated to evaluate the effect of Span and Tween on cyclopentane hydrate. The induction time describes the time taken for a noticeable crystal to form. It allows the analysis of how fast or slow it would take for hydrate to form. On the other hand, the cyclopentane hydrate crystallization time is the time taken for a complete hydrate to form and the maximum viscosity achieved. The hydrate induction time (T_i_) is estimated from Figure 3 as follows:

T_i_ = T_h_ − T_seed_ (1)

where T_h_ and T_seed_ are the time taken for hydrate to start forming and when seed where added to the mixture, respectively. The hydrate crystallization/growth time (G_t_) is calculated form Figure 2 as follows;

G_t_ = T_G_ − T_h_, (2)

where T_G_ is the time taken for complete hydrate formation.

## 3. Results and Discussion

### 3.1. Hydrate Seeds and Volume

**Figure 4:**
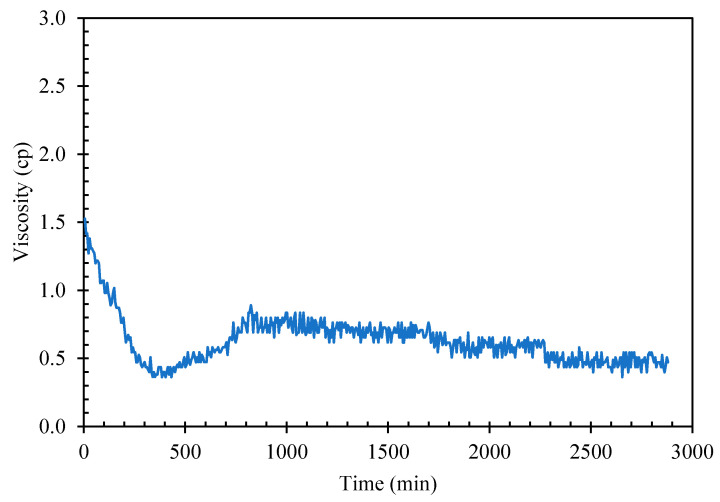
CP–Water mixture viscosity without hydrate seeds/flakes addition.

Giving sufficient time and cooling at atmospheric ambient pressure, cyclopentane would crystallize in distilled water to form solid hydrate The suspension of hydrate solids in liquid slurry increases the fluid viscosity until the point when the solid loading in the slurry mixture is packed and viscous enough to stop the rheometer spindle. The induction time, defined as the time interval from the beginning of cooling cycle to start of solid hydrate formation could be too long for practical viscosity measurement using rheometer. As seen in Figure 4, mixture of cyclopentane–distilled water (CP–water) in 50%–50% *v/v* when aged at about 2 °C for nearly 48 h, the mixture viscosity remained unchanged at around 0.5–1.0 cP with shear rate 50 1/sec indicating no hydrate formation after 48 h.

Karanjkar et al. [27] recommended to add trace of externally grown cyclopentane hydrate solids or flakes that was preprepared separately into the CP–water mixture to accelerate hydrate formation. The hydrate traces act as hydrate seeds to trigger subsequent hydrate crystallization on its surface, hence promoting the speed the hydrate to form. The quantity and shape of the hydrate seeds used should be optimized and consistent, so results from the Tween and Span effects could be compared. The induction time and hydrate crystallization time were measured repeatedly for repeatability, the standard deviation of the induction time was ±1.5, while that of the crystallization time was ±0.8. These were found to be a good agreement and consistent for further reporting.

**Figure 5:**
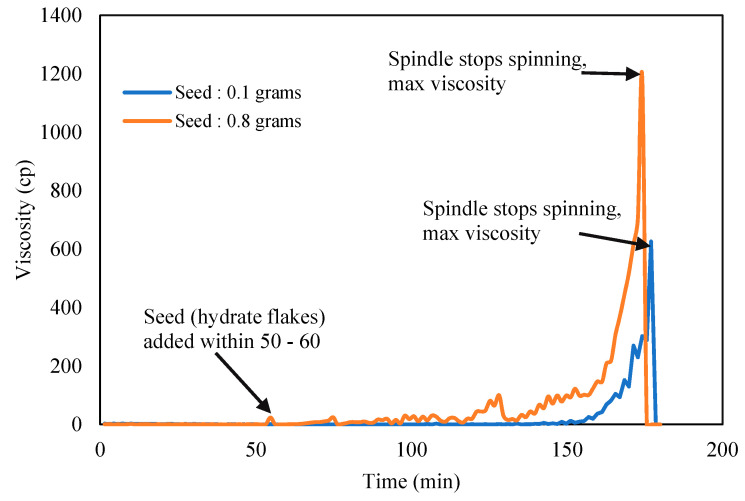
CP–water mixture viscosity with hydrate seeds/flakes addition.

Figure 5 shows the acceleration impact to growth the hydrate slurry using externally grown CP hydrate flakes. In all the experiment run, almost constant elongate shape with average length of 2 mm and 1 mm thickness was used. Seeds/flakes were added after 60 min the CP–Water mixture has attained temperature equilibrium to preset temperature at 2 °C. This was assigned as zero time for all the induction time measurement [27]. The result in Figure 5 shows that 0.1 g of hydrate flakes could produce more reliable and repeatable viscosity trend. Hence constant amount and size of flakes were then used in subsequent experiment to study surfactants effects on hydrate slurry viscosity.

### 3.2. Impact of Oil-Soluble Span 80 Surfactant on CP Hydrate Slurry Viscosity Giving

**Figure 6:**
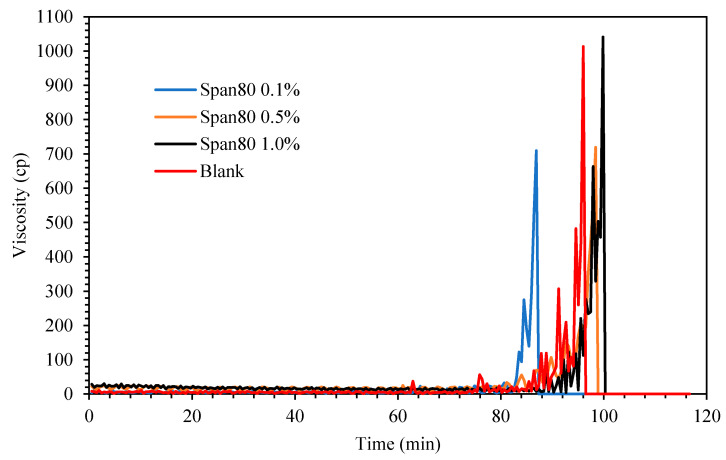
The effect of Span 80 concentration (% *v/v* in oil) on cyclopentane hydrate slurry (CP/H_2_O: 50/50; aging temperature 2 °C).

**Figure 7:**
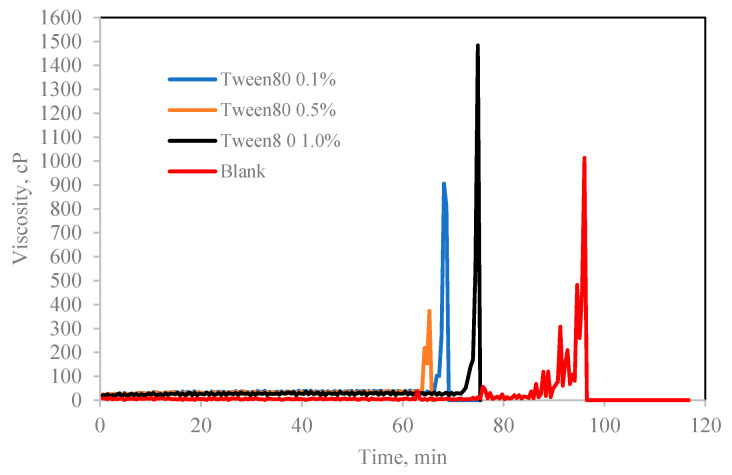
The effect of Tween 80 concentration (% *v/v* in oil) on cyclopentane hydrate slurry (CP/H2O: 50/50; aging temperature 2.0 deg C).

Figure 6 shows the viscosity changes for the cyclopentane hydrate in the presence of oil-soluble Span 80 surfactant. Exactly 0.1 g preprepared hydrate flakes was added into the cyclopentane–water mixture after 60 min the mixture was in equilibrium at 2.0 °C to initiate hydrate formation. It was noted that the viscosity trend for the blank mixture without oil-soluble surfactant Span 80 started to increase after 11 min hydrate flakes were introduced as the induction time. Its viscosity continued to progress gradually and eventually attained maximum value at about 1000 cP before the rheometer spindle ceased spinning when solid cyclopentane was completely developed in the rheometer cup holder. Subsequently the measured induction time and crystallization time (time taken to achieve maximum viscosity after induction time) for Span and Tween systems at different concentrations are presented in Table 2 and Figure 7 for extensive elaborations.

**Figure 8:**
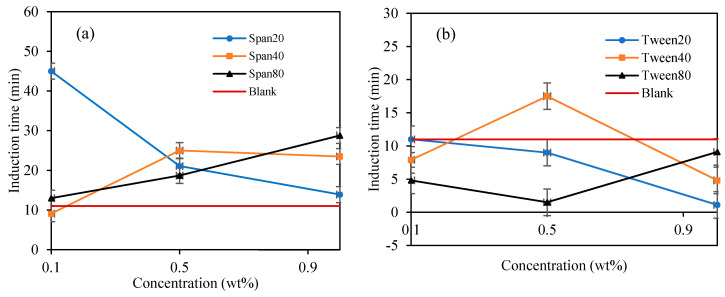
The effect of Span and Tween concentrations (% *v/v*) on CP–hydrate formation induction time. (CP/H2O: 50/50; aging temperature 2.0 deg C); (**a**) Span surfactants, (**b**) Tween surfactants.

**Figure 9:**
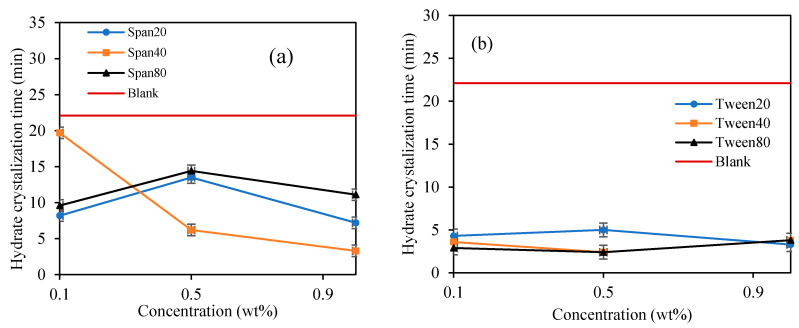
The effect of Span and Tween concentrations (% *v/v*) on CP–hydrate formation crystallization time. 50/50; aging temperature 2.0 deg C); (**a**) Span surfactants, (**b**) Tween surfactants.

When Span surfactant was added into the base mixture, it lightly emulsified the cyclopentane and water by the shearing force induced by the spinning spindle set at 50 sec^−1^ thus increasing the interfacial contact between the 2 liquid phases. [32] The results show that the Span surfactants extended the induction time for CP hydrate formation than Tween surfactants, as shown in Figure 8 and Figure 9 and Table 2. Probably due to the low HLB values of Span, which controls the intensity of emulsification of the cyclopentane and water to favor hydrate formation. This result is encouraging and confirms the potential inhibitive characteristic of non-ionic Spans against hydrate formation. The inhibitive impact of Spans and Tweens are dependent on their concentrations and HLB values.

In Figure 8a, the highest hydrate inhibition was observed in Span 20 at 0.1 wt%., however, increasing concentration significantly decreases its inhibitive impact by 69% at 1 wt%. On the other hand, the inhibitive impact of Span 40 and 80 increases with increasing concentration by 166% and 123% at 1.0 wt%. We observed that the hydrate induction time inhibitory efficiency in Span surfactants with high HLB decreases with increasing concentrations. While that of Spans with low HLB values (Span 40 and 80) increases with increasing concentration. High HLB values of surfactants exhibits high hydrophilicity and enhances the solvation of the surfactants in the mixture thus providing a perturbation effect in the water structure via hydrogen bonding. This perturbation effect prolongs the hydrate nuclei crystallization time, especially at low concentrations as demonstrated by Span 20 in Figure 8a. Contrary, the hydrophobic nature of Span with low HLB values increases with concentration, hence, increasing its inhibition impact with concentration.

In contrary, Tween which is a water-soluble non-ionic surfactant which also lightly emulsified the cyclopentane–water mixture reduced the hydrate induction time significantly compared to blank sample as shown in Figure 7 and Figure 8. Tweens generally have high HLB (hydrophilic–lipophilic balance) values compared with Spans as shown in Table 1. Thus, they tend to produce oil-in-water emulsion, i.e., oil droplets are trapped within continuous water surrounding phase. The phases configuration with water external phase promotes hydrate crystallization to occur in very short time once the hydrate flakes were added as shown in Figure 7 and Figure 8b. It was believed that the Tweens surfactant adsorbed onto the cyclopentane–water interface, leading to formation of enhanced water-wetted hydrate crystals that interacting strongly within each other with the water external phase and eventually agglomerating into larger volume and viscous hydrate slurry. Tween 20 had negligible effect on hydrate nucleation time at 0.1 wt%. The presence of Tween 40 and 80 reduced the hydrate induction time by 27% and 55%, respectively (see Figure 8b). when the concentrations of the Tweens were increased to 1.0 wt%, Tween 20 reduced the hydrate nucleation time by 91%, followed by Tween 40, 55% and that of Tween 80 was 18%. Suggesting that the maximum hydrate induction time promotion effect was observed in Tween 20 at high concentration (1.0 wt%) as shown in Figure 8b.

It was also noticed that once the hydrate crystal started to develop in the Spans and Tweens slurry systems, the maximum viscosity was attained quicker compared to the blank sample, due to the surfactant nature of Spans to enhance hydrate crystals formation [25,40,41]. Figure 9 depicts the result for time interval from the moment hydrates began to crystalline until full hydrate solid development when the rotational motion of the rotating spindle completely ceased (hydrate crystallization time). It is noticed that the Span and Tween surfactants accelerated subsequent hydrate formation once the hydrate crystallization was initiated compared to blank sample. It was suggested that emulsification by Span and Tween has increased surface contact area between cyclopentane and distilled water to encourage further agglomeration of hydrate particles in slurry and eventually complete solidification was attained [42]. Span surfactants exhibited a fast crystallization time with concentrations, while the Tween surfactants did not, as depicted in Figure 9. Probably due to the small differences in the HLB values of Tweens (see Table 1). They exhibit similar hydrate crystallization growth time. Generally, Tweens highly promote hydrate crystallization time than Spans. The average hydrate crystallization time of the blank sample was reduced by 86% and 68% by Tweens and Spans surfactants at 1.0 wt%, respectively. The best hydrate crystallization time promotional effect in the Span systems occurred at 1.0 wt%. The observed efficient hydrate crystallization time reduction in the presence of Tween and Span can be useful in other application such as hydrate-based refrigeration application, gas separations and storage [24]. However, Tween and Span has the potentials to provide hydrate slurry that can be transported safely to the surface. The findings in this study for Span 80 agrees with Liua et al. [38] who reported that the presence of Span 80 rapidly increases cyclopentane hydrate formation which results in an irregular surface growth. However, they further suggested that the effect of Span 80 on cyclopentane hinders hydrate formations and agglomerations. Karanjkar et al. [27] also confirmed the potential anti-agglomeration behavior of Span 80 at high concentrations. Hence considering Span surfactants for gas hydrate risk management in flow assurance management is recommended. Contrary to the morphology study of Dann and Rosenfeld [26], the presence of Span 20, Span 80 and Tween 65 reduced cyclopentane hydrate growth rate. However, we believe the growth rate reduction is possibly related to the ability of the surfactants to form small hydrate crystals which avoids or reduces agglomerations. On the other hand, the reduced hydrate crystallization time observed in the presence of surfactants in this study can be interpreted as the ability of the surfactants to quickly reach critical hydrate crystal size that would prevent agglomerations in the hydrate slurry.

## 4. Conclusions

Rheological measurement of viscosity for cyclopentane–water slurry hydrate with and without presence of non-ionic surfactants has been conducted in this work as varying surfactant concentrations at 2 °C in a Viscotester. The results revealed that the oil-soluble Span surfactants (Span 20, Span 40, Span 80) are potentially delaying the initialization of hydrate formation by extending the induction time for hydrate to crystalline. The best hydrate induction time inhibition impact of Span surfactants occurred in Span 20 at lower concentration (0.1 wt%). While Span 40 best prolonged the hydrate crystallization time at 0.1 wt%. However, both Span and Tween promoted cyclopentane hydrate crystallization time at all concentrations due to their surfactant behavior. Hence, confirming their potential anti-agglomeration impact via quickly achieving critical hydrate size that eliminates agglomeration and provide easy hydrate slurry that can be transported. The water-soluble Tween surfactants (Tween 20, Tween 40, Tween 80) demonstrate higher hydrate promoting impact than Span surfactants. The findings in this study will help provide insight into the rheological effect of surfactants on the hydrate slurry. The changes in fluid viscosity following hydrate slurry formation is useful information when performing fluid dynamic calculation in pipeline fluid transmission and process design for gas storage and potential application to CO_2_ gas sequestration where the rate of hydrate formation and hydrate slurry viscosity are critical.

## Figures and Tables

**Table 1:** List of chemicals used in this study.

Chemical	HLB	Density @ 25 °C (gcm^−1^)	Viscosity @ 25 °C (cp)	MW (gmol^−1^)	Supplier
Deionized water	–	1.0	0.890	18.12	Self
Cyclopentane	–	0.751	0.419	70.1	Sigma-Aldrich
Span 20	8.6	1.032	1000–2000	346.46	Sigma-Aldrich
Span 40	6.7	1.075	1000–2000	402.565	Sigma-Aldrich
Span 80	4.3	0.986	1200–2000	428.6	Sigma-Aldrich
Tween 20	16.7	1.1	370–430	1228	Sigma-Aldrich
Tween 40	15.6	1.05–1.10	400–600	1283.63	Sigma-Aldrich
Tween 80	15.0	1.06–1.09	300–500	1310	Sigma-Aldrich

**Table 2:** Measured hydrate induction time and crystallization time.

System	Conc. (wt%)	Induction Time (min) ^a^	Crystallization Time (min) ^b^
Blank	–	11	22
Span 20	0.1	45	8
	0.5	21	14
	1.0	14	7
Span 40	0.1	9	20
	0.5	25	6
	1.0	24	3
Span 80	0.1	13	10
	0.5	19	14
	1.0	29	11
Tween 20	0.1	11	4
	0.5	9	5
	1.0	1	3
Tween 40	0.1	8	4
	0.5	18	2
	1.0	5	4
Tween 80	0.1	5	3
	0.5	2	2
	1.0	9	4

^a^ Standard deviation for induction time = ±1.5; ^b^ Standard deviation for crystallization time = ±0.8.
